# The Development of an Artificial Intelligence Video Analysis-Based Web Application to Diagnose Oropharyngeal Dysphagia: A Pilot Study

**DOI:** 10.3390/brainsci14060546

**Published:** 2024-05-27

**Authors:** Chang-Won Jeong, Chung-Sub Lee, Dong-Wook Lim, Si-Hyeong Noh, Hee-Kyung Moon, Chul Park, Min-Su Kim

**Affiliations:** 1 STSC Center, Wonkwang University, Iksan 54538, Republic of Korea; mediblue@wkuh.org (C.-W.J.); cslee99@gmail.com (C.-S.L.); dwl316@wku.ac.kr (D.-W.L.); nosij123@wku.ac.kr (S.-H.N.); 2 Smart Team, Wonkwang University Hospital, Iksan 54538, Republic of Korea; 3 Institute for Educational Innovation, Wonkwang University, Iksan 54538, Republic of Korea; ybnjcw@wku.ac.kr; 4 Division of Pulmonology and Critical Care Medicine, Department of Internal Medicine, Ulsan University Hospital, Ulsan 44033, Republic of Korea; 5 Department of Regenerative Medicine, College of Medicine, Soonchunhyang University, Cheonan 31151, Republic of Korea; 6 Department of Rehabilitation Medicine, Soonchunhyang University Cheonan Hospital, Cheonan 31151, Republic of Korea

**Keywords:** artificial intelligence, critical illness, dysphagia, rehabilitation, stroke, swallowing, video

## Abstract

The gold standard test for diagnosing dysphagia is the videofluoroscopic swallowing study (VFSS). However, the accuracy of this test varies depending on the specialist’s skill level. We proposed a VFSS-based artificial intelligence (AI) web application to diagnose dysphagia. Video from the VFSS consists of multiframe data that contain approximately 300 images. To label the data, the server separated them into frames during the upload and stored them as a video for analysis. Then, the separated data were loaded into a labeling tool to perform the labeling. The labeled file was downloaded, and an AI model was developed by training with You Only Look Once (YOLOv7). Using a utility called SplitFolders, the entire dataset was divided according to a ratio of training (70%), test (10%), and validation (20%). When a VFSS video file was uploaded to an application equipped with the developed AI model, it was automatically classified and labeled as oral, pharyngeal, or esophageal. The dysphagia of a person was categorized as either penetration or aspiration, and the final analyzed result was displayed to the viewer. The following labeling datasets were created for the AI learning: oral (*n* = 2355), pharyngeal (*n* = 2338), esophageal (*n* = 1480), penetration (*n* = 1856), and aspiration (*n* = 1320); the learning results of the YOLO model, which analyzed dysphagia using the dataset, were predicted with accuracies of 0.90, 0.82, 0.79, 0.92, and 0.96, respectively. This is expected to help clinicians more efficiently suggest the proper dietary options for patients with oropharyngeal dysphagia.

## 1. Introduction

Dysphagia is the inability of a person to smoothly ingest food owing to problems with the passage of food from the mouth to the esophagus [1]. Patients with stroke, dementia, traumatic brain injury, Parkinson’s disease, and cancer are at an increased risk of developing dysphagia [2]. Dysphagia is also common in aged individuals, including those who have conditions not accompanied by swallowing-related functional or anatomical abnormalities, such as chronic obstructive pulmonary disease [3,4,5]. Notably, in a nursing care facility survey in South Korea, more than half of the patients reported having dysphagia [6].

Oropharyngeal dysphagia is characterized by difficulty initiating a swallow, and it may be accompanied by penetration, aspiration, and a sensation of residual food remaining in the pharynx [7]. Penetration, a mild form of swallowing difficulty, means that a bolus enters the laryngeal vestibule but never reaches the level of the vocal folds; this condition generally clears spontaneously [8]. Aspiration is a severe condition of dysphagia caused by accidentally inhaling food or liquid through the vocal cords into the airway [9]. The primary purpose of tube feeding for dysphagia patients is to prevent aspiration and aspiration pneumonia, which is a form of pneumonia in which a foreign body such as food, saliva, or sputum enters the alveoli and lungs through the trachea rather than the esophagus [10]. This can result in inflammation and bacterial proliferation, leading to severe complications such as sepsis, which requires invasive management and long-term care for treatment; if left untreated, it can eventually lead to death [11]. Consuming food is a basic human instinct, with tube feeding due to impaired oral feeding causing deep neck pain to patients, potentially leading to severe depression and serious complications, such as gastrointestinal bleeding, which requires rapid and accurate evaluation and management for treatment [12]. Additionally, in patients with stroke and dementia, aspiration pneumonia can lead to irreversible sequelae, from which permanent recovery is impossible. The prevalence of aspiration pneumonia, which can be as high as 20%, has been on a steep rise in recent years, with it being ranked as the fourth leading cause of death in 2016 [13]. Treating this disease using broad-spectrum antibiotics requires weeks to months [14]. Therefore, after accurately diagnosing dysphagia in a patient, a healthcare provider should determine whether to tube feed them and determine the appropriate enteral nutrition formulation for them.

The gold standard method for examining dysphagia is the videofluoroscopic swallowing study (VFSS) [15,16]. The VFSS is not the most common method of diagnosing dysphagia, but it is considered the gold standard, with the FEES (fiberoptic endoscopic evaluation on swallowing) and bedside testing also being common internationally [11]. The time required for a VFSS test is naturally greater if patient compliance is low [17]. In addition, skilled specialists take a considerable period of time to simultaneously analyze and make inferences from the large amount of data in the test results [18]. Therefore, in order to overcome these shortcomings, researchers have recently attempted to diagnose VFSS images using artificial intelligence (AI). Regarding existing studies, Kim et al. [19] collected VFSS data from 190 patients with dysphagia, selected ten frame images from the swallowing process, and applied a convolutional neural network (CNN) to classify normal swallowing, penetration, and aspiration in the VFSS. They used 665 images from 133 patients as the training dataset and 285 images from 57 patients as the testing dataset. Ten images of the swallowing process were selected, with five peak images (position of the highest hyoid bone) and five lowest peak images (position of the most inferior hyoid bone). The learning outcomes were normal (AUC = 0.942), penetration (AUC = 0.878), and aspiration (AUC = 1.000) with very high accuracies. However, their method was limited in that an entire video could not be used for the deep learning analysis. Ariji et al. [20] performed video fluorography (VFG) on twelve patients (seven men and five women). The U-Net neural network was applied to automatically segment the food material from the VFG images of both patients who swallowed normally and those who had dysphagia. For the training dataset, 1845 static images were used, which included 1005 static images of 18 swallows from three patients with healthy swallows and 840 static images of 12 swallows from two patients with aspiration or laryngeal conditions. For the validation dataset, 155 static images of six swallows from one patient with healthy swallows and 510 static images of eighteen swallows from three patients with healthy swallows were used as test dataset 1; and 1400 static images of 18 swallows from three patients with aspiration or laryngeal conditions were used as test dataset 2. The learning outcomes were high, exceeding 0.9 on the test datasets.

However, the previous studies using AI technology to diagnose patients’ swallowing disorders with VFSS videos have not examined the entire video. The limitation of these studies is that they selected several images from 300 to 500 video frames and analyzed whether there was a swallowing disorder in these images. This method is not practical in clinical practice because it requires additional effort and time for clinicians to select the appropriate individual video images. This study aimed to develop a system to solve these problems. We propose a web application that can diagnose dysphagia through a web-based database construction that handles the VFSS video data files before the evaluation and analysis, labels the generated data required for development with the artificial intelligence (AI) technology, and develops an AI model that can be applied to clinical settings based on the labeling data. In addition, the developed system was applied to stroke patients on a trial basis to investigate its accuracy and reliability.

## 2. Materials and Methods

### 2.1. Study Design and Dataset

In this study, 249 VFSS cases were randomly selected from 1348 VFSS cases, and the corresponding video clips were used to conduct a pilot study. The patients were between 25 and 96 years old (a mean age of 68.3 ± 17.8 years) and included 169 males and 80 females. Of these patients’ videos, 31 were excluded because they contained images of the patient unable to swallow food due to severe oral phase delay. In the end, 218 VFSS videos were included in developing the AI model. Aspiration was diagnosed in 141 patients by a passive VFSS video reading by neuro-rehabilitation medical doctors, and 77 patients had penetration alone.

The data were collected from patients diagnosed with dysphagia (International Classification of Diseases, 10th Revision, Clinical Modification Diagnosis Code R13.10) who were subjected to a VFSS between January 2017 and April 2022 at Wonkwang University Hospital, a 798-bed university-affiliated tertiary hospital in Iksan, South Korea. Finally, a video clip labeling dataset was created for the AI learning of the oral (n = 2355), pharyngeal (*n* = 2338), esophageal (*n* = 1480), penetration (*n* = 1856), and aspiration (*n* = 1320) datasets. Three rehabilitation physicians with more than ten years of experience in performing and reading VFSS examinations categorized all the video clips as either “normal”, “penetration”, or “aspiration”. If the specialists were not unanimous in their decision for a video, they would review it frame-by-frame till they reached a common consensus. To address potential sources of bias, the specialists and the data technicians were separated during the study periods. Regarding the data, aspiration or penetration was confirmed in 218 cases.

### 2.2. Web-Based Database to Manage VFSS Video Clips

The database schema was designed by analyzing the data generation rules and relationships of the system and considering data-based read and processing measures. The database was designed by mapping documents and classifying collections, because it used MongoDB, which is a NoSQL database.

### 2.3. Multiframe Medical Image-Labeling Web Application

**Figure 1:**
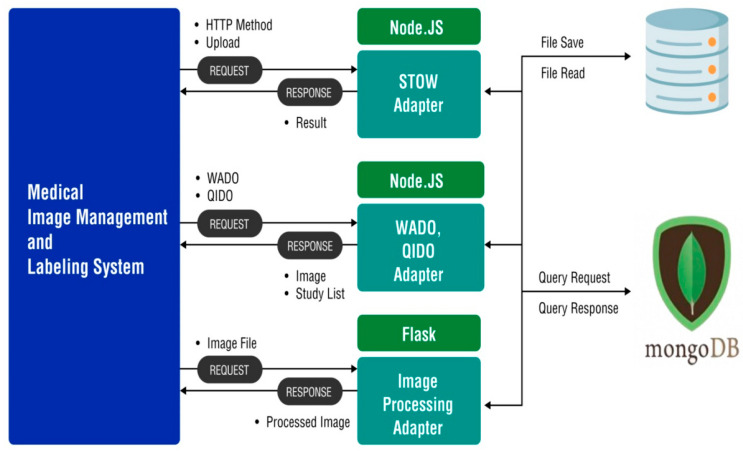
Overall system structure of the medical image-labeling web application. The overall system consists of video data management, labeling systems, web service frameworks, and storage, which consists of file systems and databases. The web platform used Node.JS, which is used to develop scalable network applications, and STOW-RS, which was used for the original image storage. WADO complies with web-based protocols for securely transmitting and sharing DICOM images over the Internet. QIDO has a function for medical image-based query requests and searches. Through Flask, a micro-web framework written in Python, the API of web applications is implemented and used to process image files. STOW-RS, STore Over the Web by Representations State Transfer; WADO, Web Access to DICOM Persistent Objects; DICOM, Digital Imaging and Communication in Medicine; QIDO, Query Based on ID for DICOM Objects; and API, Application Programming Interface.

We propose a web application for the multiframe medical image labeling. Figure 1 illustrates the overall structure of the system.

**Figure 2:**
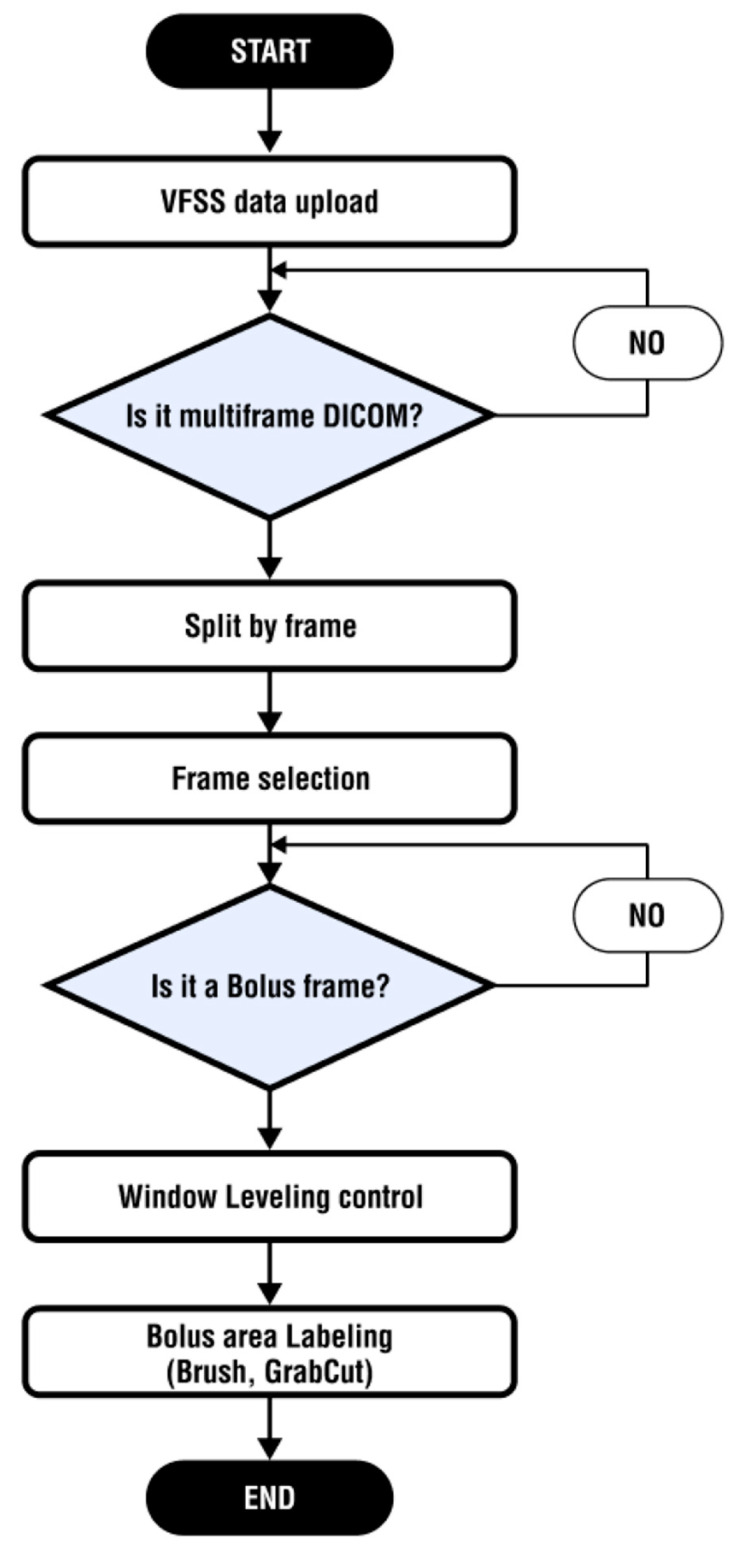
Swallowing disorder medical image-labeling process. The image data obtained through the VFSS inspection is uploaded as a DICOM file to check whether the video comprises multiple frames. The header information of the file determines this; if it is multiframe, it is automatically split by frame. The system checks for lumps of food in the selected frame. Next, it resizes the window to see the chunks of food. After that, labeling software ver. 1.0. (Brush and Grabcut) was used to start the labeling.

The proposed labeling web application manages a video file about swallowing and performs the file handling. The swallowing test images are supported in a multiframe manner to capture the entire path of the food traveling from the oral cavity to the esophagus. Compared to the cross-sectional images obtained using computed tomography or magnetic resonance imaging, multiframe images have bulky data formats with file sizes of 300 MB~1.5 GB, because they contain 200–700 images that capture movements in the body. When a file is uploaded, the node server stores the original image using the STore Over the Web (STOW) by REpresentations State Transfer (REST) Services (STOW-RSs), which separates multiple frames for labeling, transforms them into a single image, and saves the image. Subsequently, processing is performed to convert and save the audio–video interleaved file format for video analysis using the AI model. When the multiframe medical image-labeling web application requests image processing, which involves actions such as using the Brush tag for the labeling, the image is processed using the Python package in the Flask microweb framework (Figure 2).

DICOM, Digital Image and Communications in Medicine.

### 2.4. Categories of the Datasets and the Labeling Process

The three phases of swallowing include (1) the oral phase, where the food is chewed as necessary or to a consistency and form that can be swallowed before the tongue pushes the food back to induce pharyngeal swallowing; (2) the pharyngeal phase, in which a food bolus reaches the pharynx and involuntary swallowing occurs, in which the swallowing reflex causes rhythmic and involuntary contractions of various muscles in the back of the mouth, pharynx, and esophagus to push food into the pharynx and esophagus; and (3) the esophageal phase, in which the food lump is moved through the esophagus to the stomach by the peristaltic movement of the esophagus.

Each stage in which the food material (a bolus) moved from the oral phase to the pharyngeal phase and the esophageal phase was labeled, and the bolus corresponding to penetration and aspiration was labeled [20]. After defining the boundary part of each phase in advance, three technicians performed the labeling. At this time, there may have been some differences in boundary classification due to anatomical variation among each technician. In this case, the boundary classification and labeling were conducted according to the opinions of clinical experts. Oral, pharyngeal, esophageal, penetration, and aspiration were divided into five classes, and each class was labeled as oral (*n* = 2355), pharyngeal (*n* = 2338), esophageal (*n* = 1480), penetration (*n* = 1856), and aspiration (*n* = 1320).

When the VFSS file was uploaded, the server checked the multiframe image and saved it as frames with a resolution of 946 × 958 pixels. The user used the scroll bar or left and right buttons to move each image to each stage for the labeling. They then moved to the image where the food bolus was observed and adjusted the windowing values to see the food bolus more clearly. Then, the food bolus was drawn and labeled in pixel units using a brush. However, because labeling the food boli manually by pixels is not accurate, the GrabCut algorithm was used to separate the objects and backgrounds based on a designated ROI area along with the BackProjection function, which automatically selects an area with a histogram similar to the histogram of the ROI. This makes the labeling process relatively easy. A food bolus was labeled at each stage using this method. Finally, the user could export and save the segmentation in the PNG (Portable Network Graphic) format to record the labeling results. The swallowing test data were saved as a single multiframe image comprising several frames so a large number of labeling data could be stored together.

### 2.5. AI Model for Detecting Aspiration and Penetration

The VFSS videos of 218 patients were used to develop an AI model to identify airway involvement, a severe phenomenon in swallowing disorders. The AI model was tested based on the labeled data so that airway involvement could be diagnosed by dividing it into normal, aspiration, and penetration. Separately, the ability to classify the normal swallowing processes, which are divided into the oral, pharyngeal, and esophageal phases, was added to the AI model.

When the VFSS study was downloaded from the labeling web application, all the data were compressed into folders and downloaded. Any extracted compressed file contained all the labeled files in that folder and could be viewed in the form of file names (for example, Aspiration_1.3.12.2.1107.5.3.33.7367.4.202205261015030277_2_1_0020.png), separated, and saved in folders for each class, with the first token representing that class. Then, using a utility called SplitFolders, each folder was split into two folders in a ratio of 7:1:2 for the training, test, and validation sets, respectively, and used as the AI training data.

The AI training was tested in two ways. First, for a comparison with related research [15], an AI model that classified each class using EfficientNetV2 [16], which is a CNN-based classifier, was developed. Second, an AI model for object detection in each class was developed using YOLOv7 [21].

In CNN models, the learning speed typically decreases as the size of the dataset increases. However, EfficientNetV2 is a fast learning model that achieves 4 times faster training speeds and 6.8 times fewer parameters than EfficientNetV1. In addition, since the training of the EfficientNetV2 model slows down as the image size increases, the original frame size of the VFSS inspection data (946 × 958) was reduced by half (473 × 479).

Regarding the YOLOv7 model, which was adopted in this study, the original image size was inputted as the training data, and the downloaded data for the label file were the image data. These data were unavailable in the actual YOLO model; thus, the center of the contour of the image with the label file was calculated and converted to YOLO coordinates that could wrap the object from the center. The number of classes and class names were changed to match those of the current dataset, and the remaining hyperparameters were used as provided.

## 3. Results

### 3.1. Web Database System to Manage Digital Images

**Figure 3:**
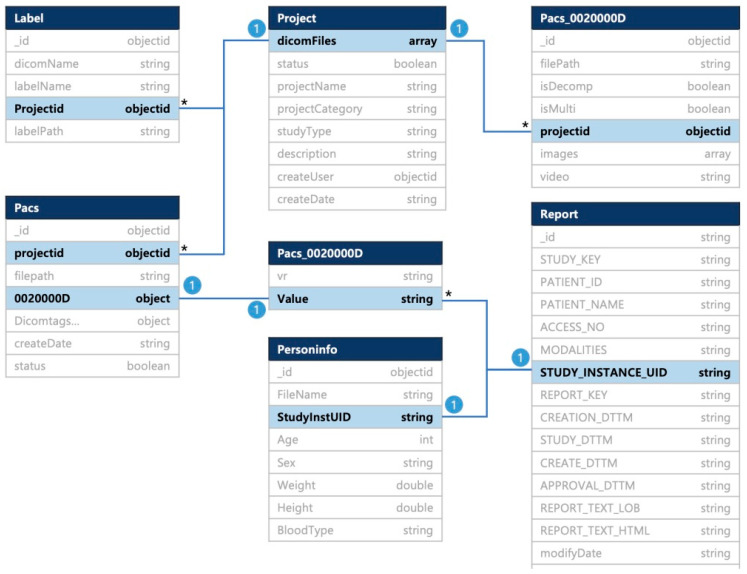
Database structure of medical image management and labeling system for diagnosis of swallowing disorders. To manage and retrieve the data for linking with the web applications, it was designed in a structure consisting of DICOM files and demographic data information. The following process was performed: project collection, where the project information was saved when adding a project; PACS collection, where the DICOM tag information was extracted and stored when a project was created or uploaded; report collection, where the clinical information of the recorded videos was saved for each study; Personinfo collection, where the demographic patient information required for this research was stored; Projectfiles collection where the file management and reference by project unit was saved; and label collection, where the report for each label that had been completed was saved. *: Multiple table values.

The database schema for the medical image management and labeling system is shown in Figure 3.

The database was designed as a project collection to store the project information when adding a project and as a Picture Archiving and Communication System (PACS) collection to extract the Digital Imaging and Communication in Medicine (DICOM) tag information when creating or uploading a project and receiving and storing the data from the self-built DICOM web server. The document of the PACS collection contained the tag information of each DICOM file; the tag information was organized in the form of an embedded document that stored each value with the value representation and the value as the key. It was designed as a report collection to manage the clinical information of the recorded videos by study unit and a PersonInfo collection to store the patient information by study unit required for the research. The “0002000D,” held in the PACS collection as an embedded document characteristic of the document database, represents the StudyInstanceUID among the values of the DICOM tag and is stored in the subvalue object. The corresponding values are referenced to the StudyInstanceUID of the PersonInfo collection, the StudyInstanceUID of the report collection, and the reference mode. Additionally, the project file collection was designed for file management and being referenced in the project collection by the projectId value (weighed). The information in the project file collection was saved when uploading a medical video file.

### 3.2. Labeling Application Software

**Figure 4:**
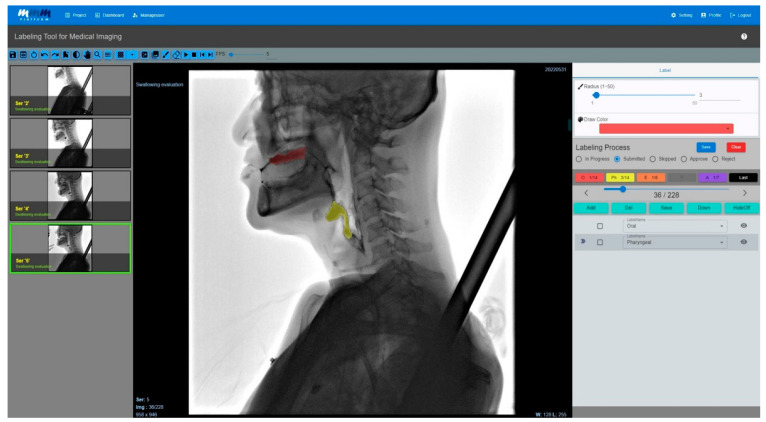
The user interface of the medical labeling web application. In order to label the multiframe image, the entire image was uploaded and divided by frame. The video was labeled as the food material progressed from the oropharyngeal to the esophageal phases. It also showed the results of the labeling images that track the food bolus as it invades the airways. In particular, there is a process for the labeling results performed by the technician to be approved by doctors specializing in swallowing disorders, designed to increase the precision of the labeling.

The complete user interface of the medical image-labeling web application is shown in Figure 4; its operation involves checking through the viewer in the labeling tool.

**Figure 5:**
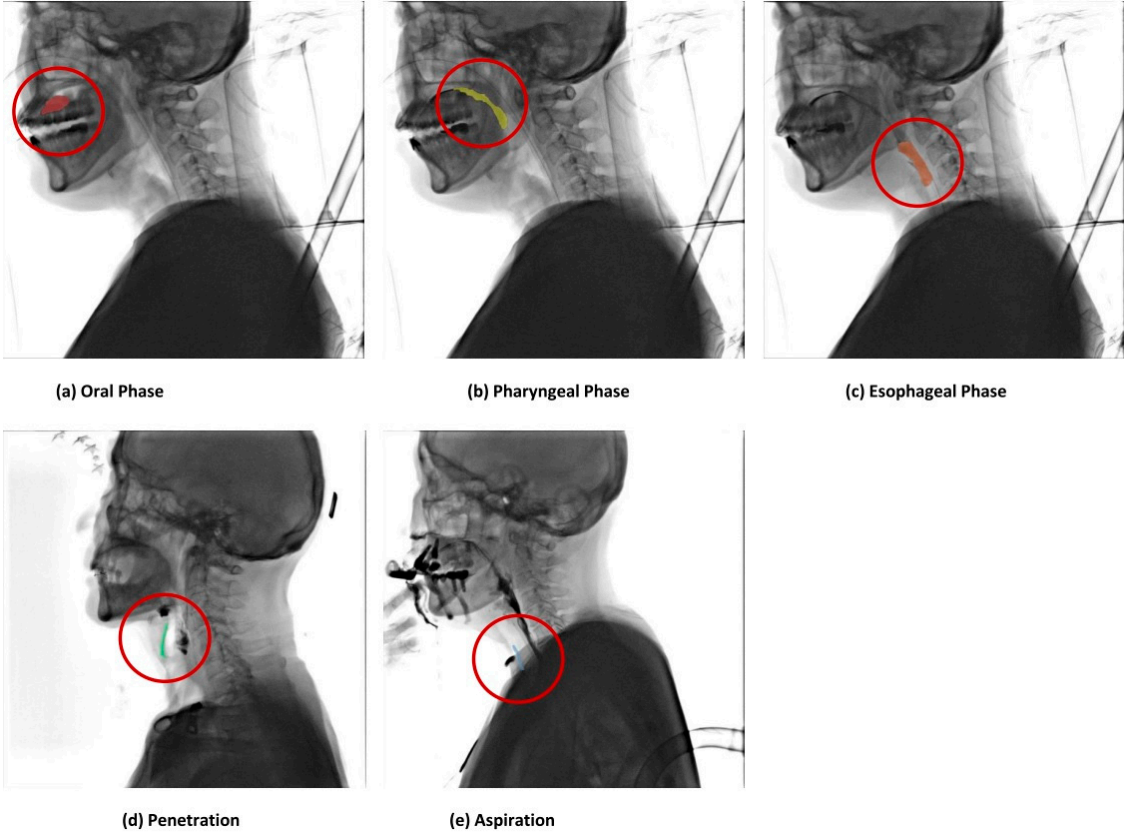
The results of labeling the food material by the user interface of the medical labeling web application. The results of labeling the food materials by the phase are shown. The results of labeling food materials in the normal state in the (**a**) oral, (**b**) pharyngeal, and (**c**) esophageal phases are shown, and the results of labeling (**d**) penetration and (**e**) aspiration in the dysphagia state are shown.

Figure 5 shows the results of labeling the food material as it moves from the pharyngeal phase to the esophageal phase and from penetration to aspiration by loading the entire image divided by frames to mark the multiframe image. In particular, a function for labelers was added to perform the labeling, and an approval process was added for the final clinician to approve the labeled data.

### 3.3. Pilot Test Results of AI Model

**Figure 6:**
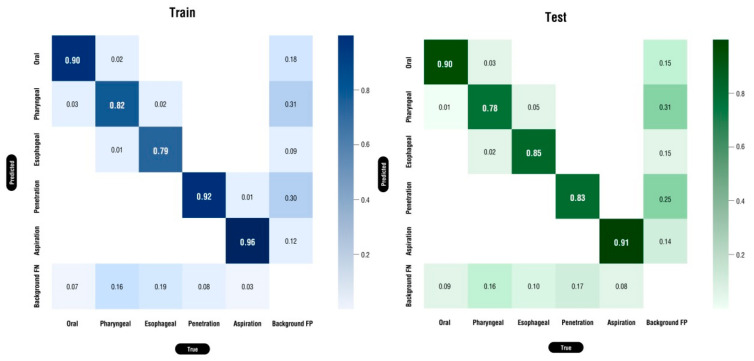
The performance evaluation results of the adopted YOLO model on the training and test data.

The results of the performance evaluation of the adopted YOLO model are shown in Figure 6.

The performance of this model was evaluated in terms of the classification accuracy using a confusion matrix. The learning results of the CNN model were inadequate compared with those obtained in previous studies. Related research has extracted ten photos of similar areas from the image of each patient [22]. A CNN finds patterns to recognize an image and uses them for classification. However, in this study, it was not easy to extract the method of the corresponding class, because various locations and food materials had been labeled [23]. Four labelers performed the labeling task. The confusion matrix revealed that there were differences in how they distinguished each area, with the adjacent areas being incorrectly recognized.

The preliminary accuracy results are presented in the training data: oral, 0.90; pharyngeal, 0.82; esophageal, 0.79; penetration, 0.92; and aspiration, 0.96. The test data showed that it was a good AI model for detecting penetration and aspiration, with an accuracy of oral: 0.90, pharyngeal: 0.78, esophageal: 0.85, penetration: 0.83, and aspiration: 0.91. The classification performance evaluation results contained the precision, recall, F1 score, and average precision of the training and testing results, as presented in Table 1.

### 3.4. AI Model Classification and Performance

**Figure 7:**
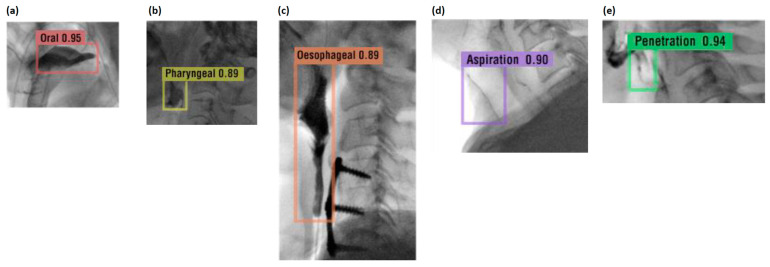
The outcome of predicting the VFSS inspection data without any training data. This figure displays the step-by-step results of the detection accuracy of the food material from the oral to the esophageal phases, penetration, and aspiration, along with the bounding box.

The AI training was tested in two ways. First, for a comparison with related studies [19], a CNN-based classifier, an AI model that classifies each class, was trained using EfficientNetV2 [16]. Then, using YOLOv7, we developed an AI model for object detection for each class [21]. As a result of experiments under similar conditions, the training time and performance of the YOLO model were proven, and the YOLO model was chosen as the leading AI engine. The model used data with the original image size and showed a good performance in terms of training time and accuracy performance. In addition, it had the advantage of being able to check the object to be diagnosed in real time in this study. In the VFSS video, it was the leading model for the real-time tracking of food masses from the mouth to the esophagus. Figure 7 shows the outcome of predicting the VFSS inspection data without any training data.

The food material was automatically detected and identified as each frame moved quickly, and Figure 7a represents the detection of the food material in the oral stage. Figure 7b shows the detection of the food material in the pharyngeal phase. Similarly, Figure 7c represents the detection of the food material in the esophageal stage, and Figure 7d shows the detection of the food material corresponding to aspiration. Finally, Figure 7e shows the detection of the food material corresponding to penetration.

**Figure 8:**
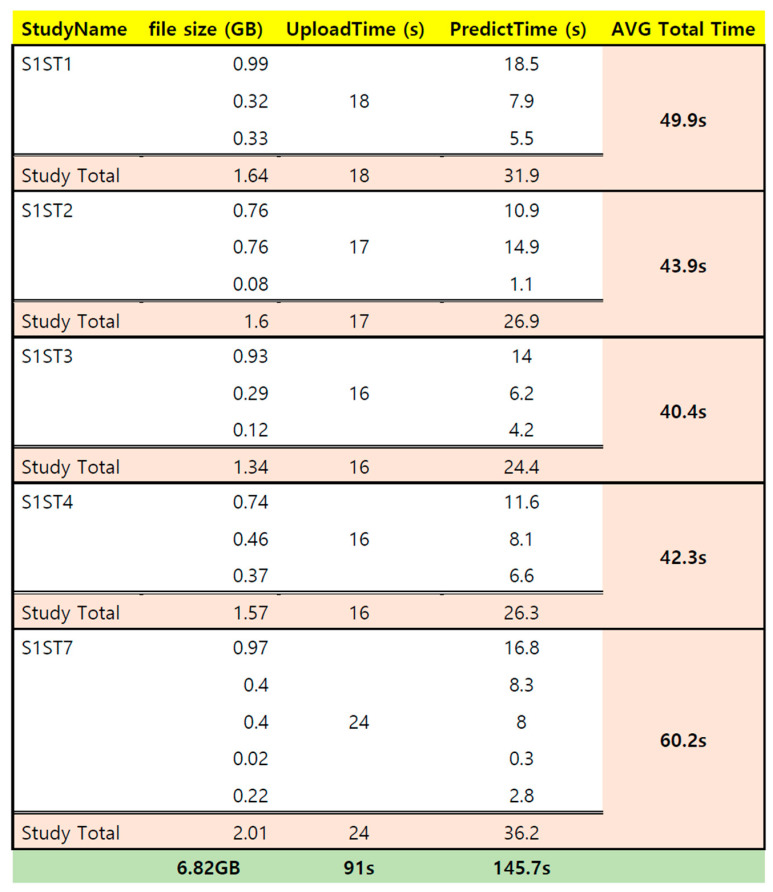
VFSS video artificial intelligence swallowing disorder diagnosis system processing time. The upload time is 16 to 24 s; the file size is 1.5 to 2 GB; and the estimated processing time to analyze by the AI model is 24 to 36 s.

The analysis time of the AI model is presented by combining the time it takes to upload and the time it takes to analyze. Unlike MRI and CT images, the VFSS images showed considerable variability depending on the patient’s cooperation with the VFSS examination, the degree of swallowing, and the time taken. Your Internet experience and PC performance can also affect the time it takes. When the AI model was used in this study, the time taken by several patients diagnosed with aspiration was displayed (Figure 8).

## 4. Discussion

This study proposes an AI web application for diagnosing aspiration or penetration in the swallowing process. An AI model that can verify the results of multiframe data generated by VFSS inspection without preprocessing or separate processing was developed for clinical applications. Labeling application software was developed to manage large amounts of data and effectively learn data generation. In particular, we aimed to reduce errors in labeling data by including clinician verification during the labeling process. Additionally, a YOLO-based model was developed to diagnose dysphagia caused by food materials, achieving an accuracy rate of at least 0.8. After optimizing modularization, the model divided the diagnosis into five classes, and the classification results were integrated into the web application.

Research on using artificial intelligence to analyze medical images has been actively published in recent years. Most of these studies used AI to diagnose diseases or investigate functions from still images, such as MRI and CT images, rather than videos. However, there are few studies that analyze video images with AI and use them to diagnose diseases. Konradi et al. [24] developed explainable artificial intelligence (XAI) to analyze Flexible Endoscopic Evaluation of Swallowing (FEES) videos. In this pilot study, it was reported that the accuracy of the training data was 0.925 and the testing data was 0.571 to diagnose swallowing disorders [24]. Similar to this study, Jeong et al. [25] attempted to diagnose swallowing disorders using VFSS video with the ResNet3D AI model. Multiple indicators, including the oral phase duration, pharyngeal delay time, pharyngeal response time, and pharyngeal transit time, were used, and the accuracy was reported to be 0.901–0.981. Medical images stored as videos (e.g., echocardiography, gastroscopy, fetal ultrasound, gait analysis, etc.) make it difficult to diagnose diseases using AI. This study is one of a few studies in which an AI automatically analyzes swallowing disorders after uploading the entire video file. Research on diagnosing diseases using artificial intelligence from medical videos is still in its early stages, and suitable AI models are also in the experimental stage. However, medical video AI research is expected to continue to be active.

Regarding our study, the average time taken to detect aspiration and penetration using the proposed AI model using EfficientNetV2 and YOLOv7 for the entire VFSS video was 40–60 s (700 frames). The model also exhibited an accuracy of 90% in diagnosing aspiration and penetration. Regarding the difficulties faced in using a VFSS, it is time-consuming for a doctor to examine and accurately read a VFSS because it often requires multiple video views. Stroke patients that are subjected to a VFSS often also have hemiplegia or quadriplegia, as well as dysphagia; therefore, the long waiting time in the clinic to obtain their medical results puts a lot of pressure on them. Additionally, the VFSS has the limitation of poor inter- and intra-rater reliability [16,22,26]. Inexperienced examiners often misinterpret VFSS results because of the complex anatomy of the human neck and the poor video quality that arises from attempting to record uncooperative patients [16]. One study showed an inter-rater reliability coefficient of 0.9 between well-trained and experienced examiners and an inter-rater reliability coefficient of 0.6 between less skilled and experienced examiners [26]. The AI model developed in our study to diagnose airway aspiration and penetration from an entire VFSS video can contribute to reducing patient waiting times and improving the reliability and validity of testing [27].

In our study, four labelers labeled food material in the swallowing process of multiframe data to diagnose dysphagia through the food material in the VFSS video of patients with dysphagia. However, if the food material overlapped ambiguously between sections, it was labeled differently from the perspective of the labeler, resulting in poor learning results. To solve this problem, rehabilitation physicians were tasked with manually reading the VFSS test and reviewing whether the labelers had labelled it properly. Additionally, the accuracy of the labeling training data was improved through verification.

This study has several limitations. Since the system was developed for patients in a single institution, additional validation tests are required on patients from external hospitals. Compared to previous studies, we used many patients’ VFSS videos to develop AI models, but more VFSS videos need to be trained on this model to be more reliable. In addition, many diseases cause swallowing disorders, and this study did not consider the characteristics of disease-specific swallowing disorders. Airway invasion, such as aspiration or penetration, has the most important diagnostic value in dysphagia. Nevertheless, depending on the disease, there may be differences in how it manifests itself. The optimization of AI VFSS video diagnostics by cause of swallowing disorders will be further implemented in future studies.

## 5. Conclusions

This study presents an AI model that can analyze an entire VFSS video to diagnose the swallowing disorder of a patient. To achieve this goal, a web database system was built to manage extensive multiframe data and multiframe medical image-labeling web software. This system could analyze entire frames in a VFSS inspection video and perform machine learning. Using the developed AI model, a pilot test was performed using a partial dataset that showed a high sensitivity for detecting aspiration and penetration in the oropharyngeal phase. The use of artificial intelligence to accurately analyze swallowing disorders within tens of seconds of the entire VFSS video, rather than just a few images, increases the possibility of using this technology in clinical practice. We believe that the proposed study can encourage future research on using AI to diagnose diseases and conditions in patients through real-time medical video examinations.

## Figures and Tables

**Table 1:** The classification performance evaluation results.

**Train**				
**Class**	**Precision**	**Recall**	**F1 Score**	**mAP@.5**
All	0.783	0.791	0.786	0.835
Oral	0.829	0.849	0.838	0.914
Pharyngeal	0.772	0.662	0.712	0.789
Esophageal	0.785	0.745	0.764	0.734
Penetration	0.745	0.788	0.765	0.831
Aspiration	0.787	0.91	0.844	0.907
**Test**				
**Class**	**Precision**	**Recall**	**F1 Score**	**mAP@.5**
All	0.71	0.793	0.749	0.781
Oral	0.806	0.832	0.818	0.857
Pharyngeal	0.608	0.702	0.651	0.688
Esophageal	0.68	0.81	0.739	0.753
Penetration	0.678	0.757	0.715	0.76
Aspiration	0.776	0.865	0.818	0.844

mAP@.5, mean Average Precision at 0.5. The object detection model implies the average of the Average Precision for all classes in the case where the threshold is 0.5.

## Data Availability

The datasets analyzed during the current study are available from the corresponding author on reasonable request. The data are not publicly available due to privacy or ethical restrictions.
